# Comparison of prewarming plus intraoperative warming with intraoperative warming alone in patients undergoing minimally invasive thoracic or abdominal surgery: A systematic review and meta-analysis

**DOI:** 10.1371/journal.pone.0310096

**Published:** 2024-09-16

**Authors:** Ni Ding, Jingjing Yang, Cuiying Wu

**Affiliations:** 1 Operating Room, Ningbo Medical Center LiHuiLi Hospital, Ningbo, Zhejiang, China; 2 Department of Cardiac Vascular Surgery, Ningbo Medical Center LiHuiLi Hospital, Ningbo, Zhejiang, China; IRCCS: IRCCS Ospedale San Raffaele, ITALY

## Abstract

**Objective:**

Prewarming has been recommended to reduce intraoperative hypothermia. However, the evidence is unclear. This review examined if prewarming can prevent intraoperative hypothermia in patients undergoing thoracoscopic and laparoscopic surgeries.

**Methods:**

PubMed, CENTRAL, Web of Science, and Embase databases were searched for randomized controlled trials (RCTs) up to 15^th^ January 2024. The primary outcome of interest was the difference in intraoperative core temperature. The secondary outcomes were intraoperative hypothermia (<36°) and postoperative shivering.

**Results:**

Seven RCTs were eligible. Meta-analysis showed that intraoperative core temperature was significantly higher at the start or within 30mins of the start of the surgery (MD: 0.32 95% CI: 0.15, 0.50 I^2^ = 94% p = 0.0003), 60 mins after the start of the surgery (MD: 0.37 95% CI: 0.24, 0.50 I^2^ = 81% p<0.00001), 120 mins after the start of the surgery (MD: 0.34 95% CI: 0.12, 0.56 I^2^ = 88% p = 0.003), and at the end of the surgery (MD: 0.35 95% CI: 0.25, 0.45 I^2^ = 61% p<0.00001). The incidence of shivering was also significantly lower in the prewarming group (OR: 0.18 95% CI: 0.08, 0.43 I^2^ = 0%). Prewarming was also associated with a significant reduction in the risk of hypothermia (OR: 0.20 95% CI: 0.10, 0.41 I^2^ = 0% p<0.0001). The certainty of the evidence assessed by GRADE was “moderate” for intraoperative core temperatures at all time points and “low” for minimal intraoperative core temperature, shivering, and hypothermia.

**Conclusion:**

Moderate to low-quality evidence shows that prewarming combined with intraoperative warming, as compared to intraoperative warming alone, can improve intraoperative temperature control and reduce the risk of hypothermia and shivering in patients undergoing thoracoscopic and laparoscopic procedures.

## Introduction

Hypothermia, defined as core body temperature <36°C, is a common complication seen during surgical procedures. It mainly occurs due to the action of anesthetic drugs on the patient’s thermoregulatory system combined with reduced metabolism [1]. Other factors like loss of heat from cutaneous surfaces, temperature of the operating room, and opening of the surgical planes leading to increased loss of surface heat also contribute to hypothermia [2]. A study has shown that the incidence of hypothermia can be as high as 44.3% [3]. Most patients receive only passive warming using surgical draping, sheets, or cotton blankets, and only about 14% of patients receive active warming using space heaters, electric heaters, or electronic blankets [3]. Intraoperative hypothermia has been shown to increase postoperative complications like surgical site infections, increased bleeding, transfusion requirement, drug metabolism disorders, shivering, and cardiovascular events [3–6]. It can also contribute to higher mortality rates, increased readmission rates, and longer length of hospital stay [7]. Several risk factors for hypothermia that have been identified include age, body mass index, baseline core body temperature, irrigation fluids, total blood loss, duration of surgery, and, most importantly, the type of surgery [3, 8].

In the current era, minimally invasive surgery like thoracoscopic and laparoscopic surgery is gaining popularity due to small incisions, shorter recovery time, and early hospital discharge, leading to reduced medical costs [9, 10]. Compared to open procedures, minimally invasive surgery does not involve extensive exposure of tissue and may, therefore, result in reduced heat loss [8]. Therefore, the potential risk of hypothermia in such surgeries must be studied separately and not combined with open procedures. To reduce the risk of hypothermia, a prewarming strategy has been suggested. This involves using active heating devices before induction of anesthesia to maintain body temperature. Prewarming can increase peripheral tissue temperature, thereby reducing the central-to-peripheral temperature gradient and preventing thermal redistribution. Research on open surgical procedures has shown that prewarming may reduce the risk of hypothermia, but evidence on minimally invasive surgeries is limited [11, 12]. This review examined evidence on the efficacy of prewarming in addition to intraoperative warming on outcomes of thoracoscopic and laparoscopic surgeries.

## Material and methods

### Criteria for selection

Randomized controlled trials (RCTs) published in full-text and examining the association between prewarming and intra-operative core temperatures in patients undergoing minimally invasive thoracic or abdominal surgeries were eligible. Inclusion criteria in PICOS format were as follows:

*Population*: Adult patients undergoing any type of laparoscopic or thoracoscopic surgery.

*Intervention*: Prewarming before surgery by any modality with intra-operative warming.

Comparison: Only intraoperative warming.

Outcomes: Intra-operative core temperature, shivering, hypothermia.

We excluded 1. studies not on laparoscopic or thoracoscopic surgery, 2. observational studies, 3. studies not reporting relevant outcomes, 4. non-English language studies, and 5. unpublished data and abstracts.

The primary outcome of interest was the difference in intraoperative core temperature. The secondary outcomes were intraoperative hypothermia (<36°C) and postoperative shivering.

### Identification of studies

The review protocol was preregistered on PROSPERO (Registration number CRD42024498037). Four online databases, PubMed, CENTRAL, Web of Science, and Embase, were independently searched by two reviewers. The keywords used were: “minimally invasive surgery,” “thoracoscopic,” “laparoscopic,” “prewarming,” and “hypothermia.” Details of the search strategy are shown in S1 Table. No filter was applied for language, publication time, and study design. Studies published up to 15^th^ January 2024 were included. Google Scholar was explored as a source of gray literature for any missed studies. Authors of abstracts were contacted for complete information when required. Bibliographic data of recent review articles were also scanned for any pertinent studies.

### Selection of studies

The review was based on the PRISMA statement reporting guidelines (S2 Table) [13]. All records were downloaded, imported, and deduplicated using the software EndNote. The two reviewers conducted an inclusive screening to exclude non-relevant articles. All selected studies were further reviewed by examining their titles and abstracts and refined to include only those relevant to the review. Full texts of the selected articles were screened further. The two reviewers examined each article based on inclusion criteria. Articles with completely overlapping articles were excluded. In case of incomplete information, the corresponding author was contacted once via email. Studies with missing data were not included in the meta-analysis. All discordance between reviewers was resolved by consensus. The bibliography of eligible articles was hand-searched for additional articles.

### Data management and study quality

Two independent reviewers extracted information on the author’s last name, year of publication, study location, surgery type, anesthesia used, study group sample size, duration of surgery, baseline temperature, prewarming time, prewarming system used, method of intraoperative warming, operation theatre temperature and humidity, and outcome data.

Two reviewers examined The risk of bias in all RCTs using the Cochrane Collaboration risk of bias-2 tool [14]. The RCTS were assessed for random sequence generation and allocation concealment, deviation from intended intervention, missing data, measurement of outcomes by blinding, selection of reported results, and overall risk of bias. Each item was rated as “high risk,” “low risk, or “some concerns.” Certainty of evidence was also examined using the GRADE (Grading of Recommendations Assessment, Development and Evaluation) approach.

### Statistical analysis

All statistical analyses were done using “Review Manager” (RevMan, version 5.3; Nordic Cochrane Centre (Cochrane Collaboration), Copenhagen, Denmark; 2014). All outcome data from the studies were extracted in a tabular form. Intraoperative temperature data were segregated based on the time of measurement. Five time points were defined for the meta-analysis: T1: at anesthesia induction or within 10mins of induction, T2: start of the surgery or within 30mins of the start of surgery, T3: 60mins after the start of the surgery, T4: 120mins after the start of the surgery, and T5: end of the surgery. Mean values of core temperatures were pooled at these time points to obtain Mean Difference (MD) and 95% confidence intervals (CI). Data on shivering and hypothermia were pooled to calculate odds ratios (ORs). Meta-analysis was done using the random-effect model due to the methodological heterogeneity between studies. Funnel plots were not drawn due to a limited number of studies. The I^2^ statistic was used to determine inter-study heterogeneity. I^2^ <50% meant low, and >50% meant substantial heterogeneity. Subgroup analysis was conducted based on surgery type, abdominal vs thoracic surgery. A sensitivity analysis was conducted to assess the effect of individual studies on the outcome by removing one study at a time and re-examining the results.

## Results

**Fig 1:**
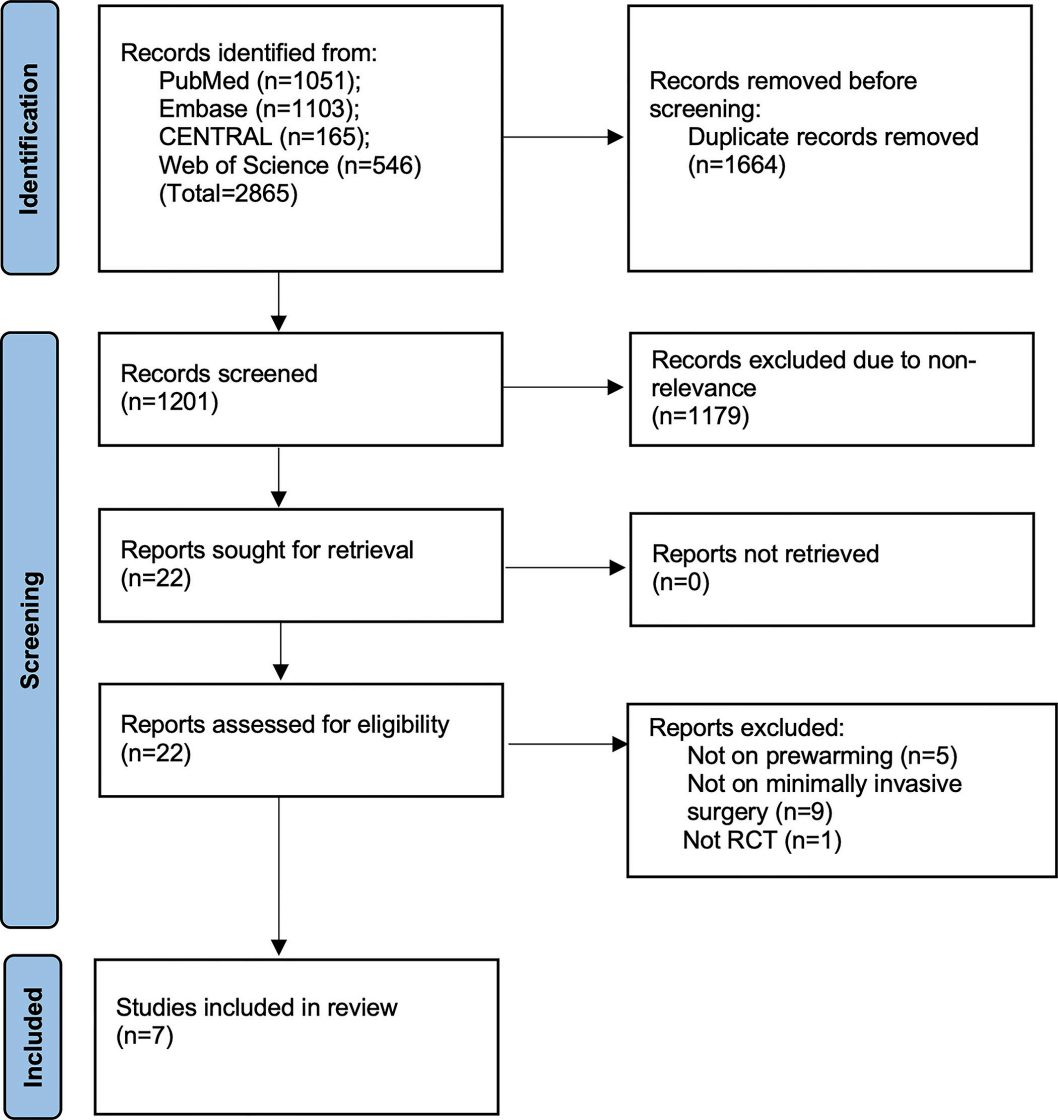
Study flowchart.

Records obtained during the literature search are shown in Fig 1.

A literature search identified 2865 articles. Duplicates were removed, leaving 1201 unique results. The reviewers examined the articles for primary eligibility and 22 studies that were selected for the full-text analysis underwent detailed examination. Finally, seven studies were included in the analysis [15–21]. List of excluded studies is presented as S3 Table. Characteristics of the studies are shown in Table 1.

**Table 1:** Research characteristics of meta-analysis.

Study	Location	Anesthesia	Surgery type	Groups	Sample size	Operating time	Baseline temperature	Prewarming time*	Prewarming system	Intra-op warming	OT temperature & humidity
Shirozu 2023 [16]	Japan	GA with epidural	Laparoscopic	Prewarming Control	52 52	273± 114 303± 163 (mins)	37± 0.4 37.1 ± 0.4	NR	Forced air warming system with blanket	Forced air warming system with blanket	25–28, 40–50%
Zhang 2022 [15]	China	GA	Laparoscopic resection of colorectal cancer	Prewarming Control	35 35	3.8± 0.7 3.7± 0.5 (hrs)	36.5± 0.09 36.5± 0.16	≥20 mins	Forced air warming blanket	Forced air warming blanket	21–23, 40–60%
Li 2022 [17]	China	GA	Thoracoscopic	Prewarming Control	84 121	NR	NR	30 mins	Forced air warming	Heating blanket	NR
Xiao 2020 [20]	China	GA	Thoracoscopic	Prewarming Control	49 49	126.6± 24 135.4± 28.7 (mins)	NR	30 mins	Forced air warming blanket	Upper body forced air warming blanket	22± 1, 40–60%
Lee 2020 [21]	Korea	GA	Gynecologic laparoscopic	Prewarming Control	25 26	61.3 ± 16.4 61.1 ± 16.2 (mins)	NR	10 mins	Forced air warming device	Forced air warming blanket over upper trunk and extremities	21–22, NR
Witte 2010 [19]	Belgium	GA	Laparoscopic colorectal surgery	Prewarming Control	9 8	114± 42 128± 47 (mins)	NR	30 mins	Forced air warming blanket	Forced air warming blanket	20, NR
Camus 1995 [18]	France	GA	Laparoscopic cholecystectomy	Prewarming Control	8 8	NR	37.1± 0.1 37.1± 0.1	60 mins	Forced air warming blanket	Cotton sheet	NR

NR, not reported; GA, general anesthesia; OT, operation theatre. Data for this table was extracted by authors ND and JY. The authors confirm that all the above studies were eligible for the systematic review.

*prewarming was done before anesthesia induction in all studies.

The RCTs originated from China, Japan, Korea, Belgium, and France. Three studies reported data on thoracoscopic surgeries, while the remaining four were on laparoscopic surgeries. All patients received general anesthesia for the procedures. The sample size varied from 16 to 205. Prewarming was done using forced air warming in all studies. However, the duration varied from 10 to 60 minutes. The operating theatre temperature ranged from 20 to 28°C. The risk of bias analysis is presented in Table 2. None of the studies had a high risk of bias.

**Fig 2:**
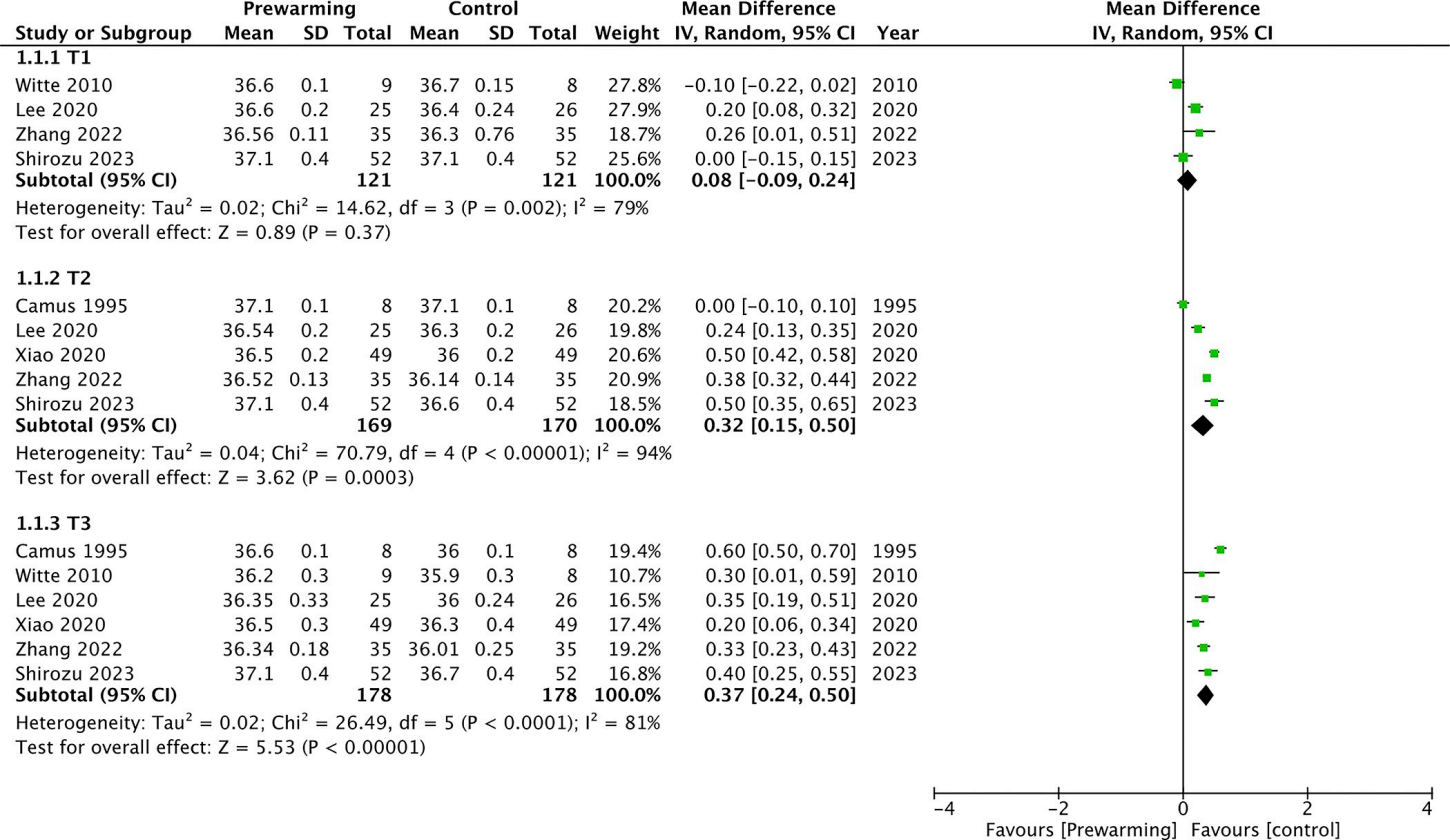
Meta-analysis of intraoperative core temperatures between prewarming and control groups at T1, T2, and T3.

**Fig 3:**
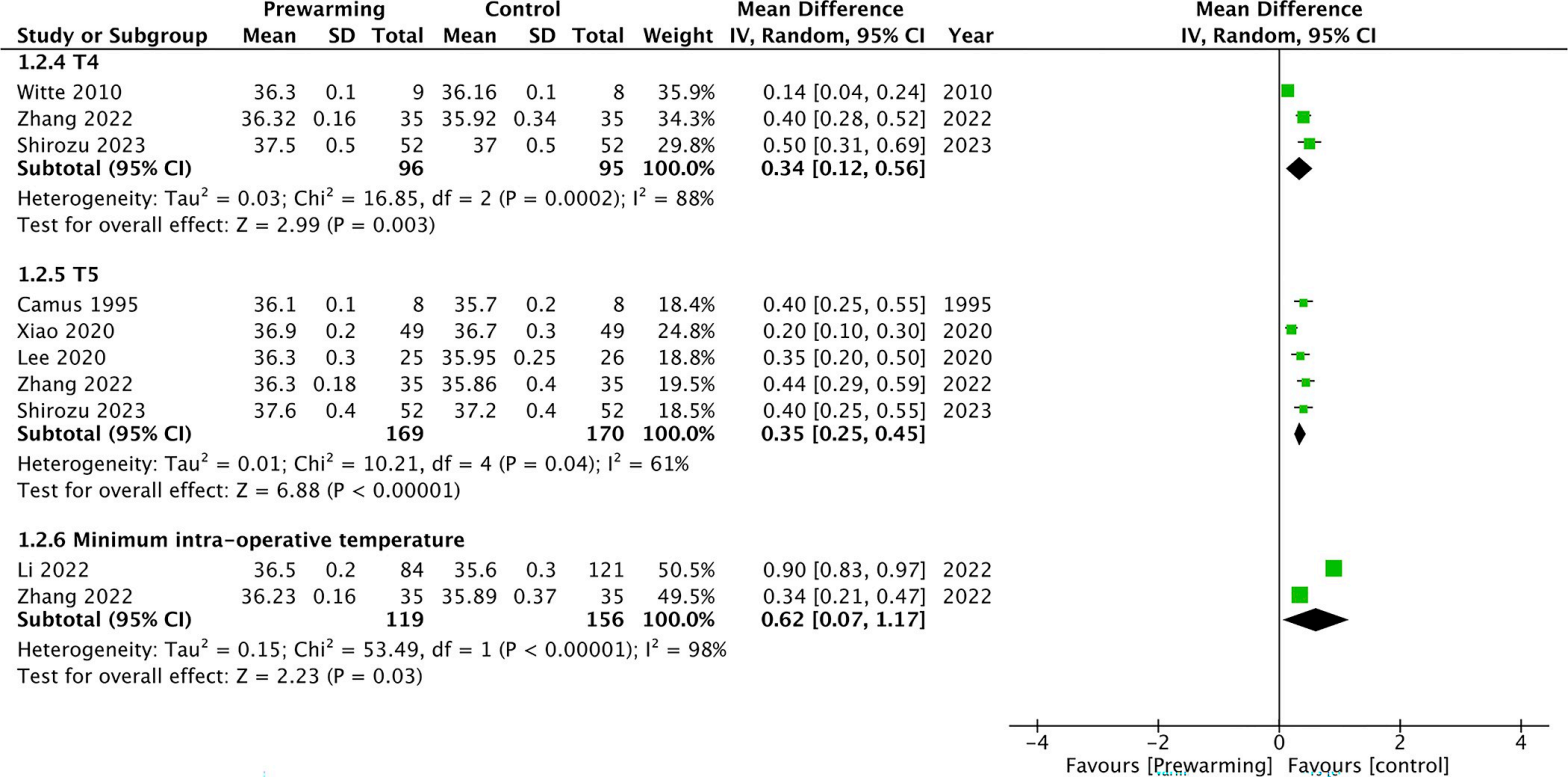
Meta-analysis of intraoperative core temperatures between prewarming and control groups at T4, T5, and minimal intra-operative temperature.

Meta-analyses for intraoperative core temperatures between prewarming and control groups are demonstrated in Figs 2 and 3.

**Table 2:** Risk of bias analysis.

Study	Randomization process	Deviation from intended intervention	Missing outcome data	Measurement of outcomes	Selection of reported result	Overall risk of bias
Shirozu 2023 [16]						
Zhang 2022 [15]						
Li 2022 [17]						
Xiao 2020 [20]						
Lee 2020 [21]						
Witte 2010 [19]						
Camus 1995 [18]						

Green box: Low risk of bias

Orange box: Some concerns

**Fig 4:**
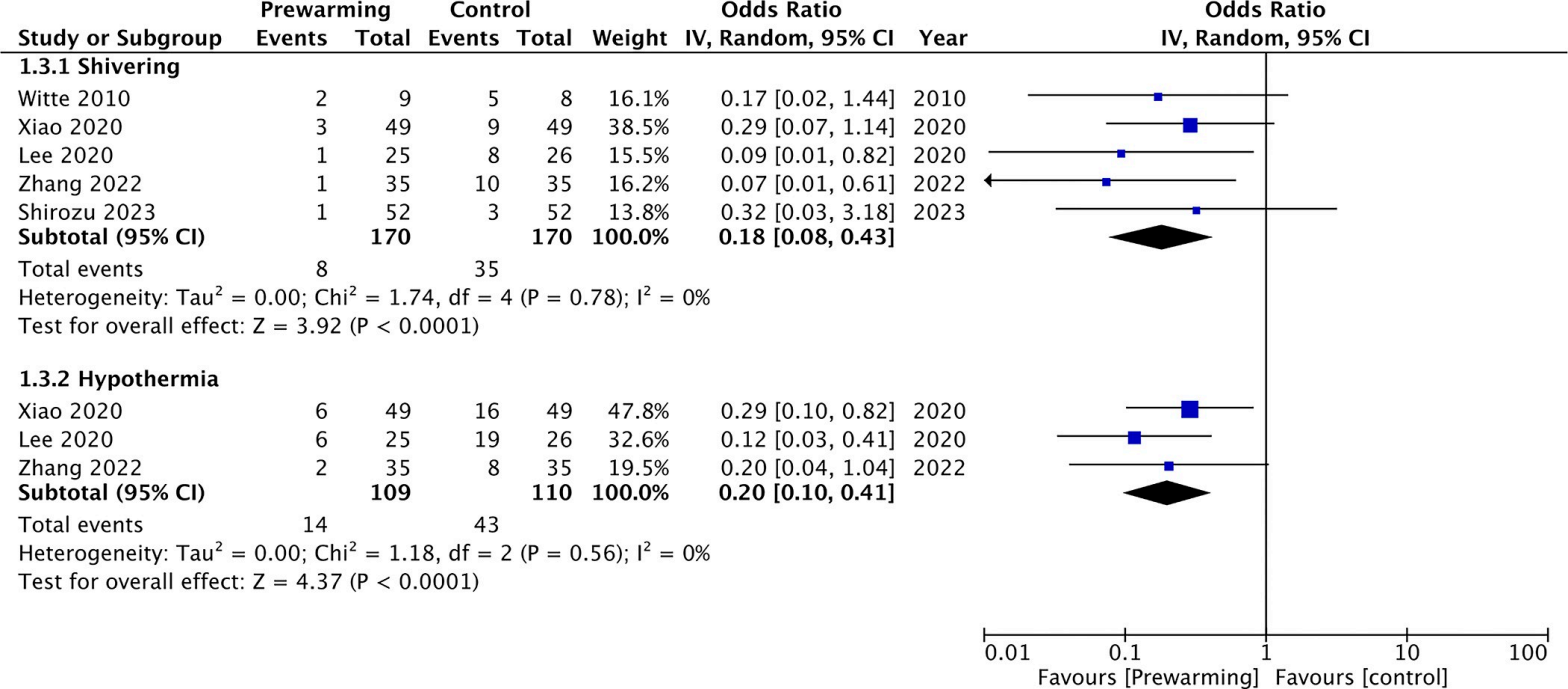
Meta-analysis of shivering and intra-operative hypothermia.

There was no statistically significant difference in core temperatures at T1 (MD: 0.08 95% CI: -0.09, 0.24 I^2^ = 79% p = 0.37). However, core temperature at T2 (MD: 0.32 95% CI: 0.15, 0.50 I^2^ = 94% p = 0.0003) and T3 (MD: 0.37 95% CI: 0.24, 0.50 I^2^ = 81% p<0.00001) were significantly higher in the prewarming group. Similarly, meta-analysis showed that intraoperative core temperature at T4 (MD: 0.34 95% CI: 0.12, 0.56 I^2^ = 88% p = 0.003) and T5 (MD: 0.35 95% CI: 0.25, 0.45 I^2^ = 61% p<0.00001) were significantly higher in the prewarming group. The minimal intra-operative core temperature was also significantly higher in the prewarming group (MD: 0.62 95% CI: 0.07, 1.17 I^2^ = 98% p = 0.03). The incidence of shivering was significantly lower in the prewarming group (OR: 0.18 95% CI: 0.08, 0.43 I^2^ = 0% p<0.0001). Prewarming was also associated with a significant reduction in the risk of hypothermia (OR: 0.20 95% CI: 0.10, 0.41 I^2^ = 0% p<0.0001) (Fig 4). On sensitivity analysis, there was no change in significance of any outcome on exclusion of any included study.

Subgroup analysis was conducted based on the surgery type (Table 3). The significance of the results did not change for abdominal and thoracic surgery groups for all outcomes. The GRADE assessment of evidence is shown in S4 Table. The certainty of the evidence was “moderate” for intraoperative core temperatures at all time points and “low” for minimal intraoperative core temperature, shivering, and hypothermia.

**Table 3:** Subgroup analysis based on surgery type.

Outcome	Group	Number of studies	Mean Difference [95% confidence intervals]	I^2^
Intraoperative core temperature at T1	Abdominal Thoracic	4 0	0.08 [-0.09, 0.24] -	79 -
Intraoperative core temperature at T2	Abdominal Thoracic	4 1	0.28 [0.08, 0.48] 0.50 [0.42, 0.58]	94 -
Intraoperative core temperature at T3	Abdominal Thoracic	5 1	0.41 [0.28, 0.54] 0.20 [0.06, 0.34]	77 -
Intraoperative core temperature at T4	Abdominal Thoracic	3 0	0.34 [0.12, 0.56] -	88 -
Intraoperative core temperature at T5	Abdominal Thoracic	4 1	0.40 [0.32, 0.47] 0.20 [0.10, 0.30]	0 -
Minimum intra-operative temperature	Abdominal Thoracic	1 1	0.34 [0.21, 0.47] 0.90 [0.83, 0.97]	- -
**Outcome**	**Group**	**Number of studies**	**Odds ratio [95% confidence intervals]**	**I** ^**2**^
Shivering	Abdominal Thoracic	4 1	0.14 [0.05, 0.40] 0.29 [0.07, 1.14]	0 -
Hypothermia	Abdominal Thoracic	2 1	0.14 [0.05, 0.39] 0.29 [0.10, 0.82]	0 -

## Discussion

About 90% of heat loss noted during surgical anesthesia occurs from the patient’s cutaneous surface to the environment. As a preventive measure, several cutaneous warming techniques, such as passive warming using surgical drapes, cotton blankets, or active warming by warmed air and water have been suggested [7]. Evidence from a Cochrane review [6] of 69 RCTs has shown that active warming is more effective in maintaining the patient’s core temperature perioperatively as compared to passive warming alone. Also, the use of active warming systems leads to a significant reduction in hypothermia-associated complications, including surgical site infections, shivering, and blood loss. Current guidelines by the Association of Perioperative Registered Nurses, 2009, suggest that patients should be prewarmed using any active cutaneous warming system to further improve outcomes [12]. It includes warming of the patient’s peripheral tissues or skin surfaces before induction of anesthesia [11, 12]. Prewarming can be done either in the anesthesia preparation area or just before the induction of anesthesia in the operating room itself.

A study by de Brito et al. [12] examined evidence on the efficacy of prewarming by reviewing 14 studies on different surgical procedures and concluded that forced-air warming was an effective method in reducing the risk of hypothermia. A study by Lau et al. [22] used a mixed cohort of non-cardiac surgery patients to show that at least 30 minutes of prewarming reduces the risk of intraoperative hyperthermia. Horn et al [23] have shown that the duration of prewarming does not affect outcomes. In an RCT of 200 patients undergoing 60–90 min surgical procedures, patients were divided into four groups of 10, 20, and 30 minutes of prewarming or passive insulation only (the control group). Around 69% of patients in the control group developed hypothermia, whereas only 13%, 7%, and 6% of patients developed hypothermia after 10, 20, and 30 minutes of prewarming, respectively. Similarly, Becerra et al. [24], in an RCT of patients undergoing prostatic transurethral resection, have shown that 15, 30, or 45 mins of prewarming results in better intraoperative temperature control with reduced risk of hypothermia and postoperative complications. Nevertheless, there have been reports in the literature which do not support the use of prewarming. In a recent RCT, Recio-Pérez et al [25] have shown that prewarming with a convection air device did not lead to significantly higher intraoperative core temperatures with no difference in the incidence, duration, and magnitude of hypothermia. The authors suggested that strict intraoperative temperature control measures, early initiation of warming, and routine monitoring for hypothermia were more essential than prewarming.

Given the contrasting evidence in the literature, the current review examined the effectiveness of prewarming in a very specific cohort of patients. Data was collated from seven RCTs focusing only on commonly practiced thoracoscopic and laparoscopic surgeries to obtain high-quality evidence. Our review found that there was a consistent statistically significant difference in the intraoperative core temperatures at all time points from the start of the surgery till its end. An MD of temperature ranging from 0.32° to 0.37°C was noted between the prewarming and the control groups. Visual inspection of forest plots also revealed that the results of individual studies were consistently in favor of prewarming as compared to intraoperative warming alone. The risk of intraoperative hypothermia in the prewarming group was 12.84%, while that of the control group was 39.09%, with a statistically significant difference. Meta-analysis also showed that the risk of shivering was reduced by 82% by using prewarming. Just 4.70% of patients in the prewarming group experienced shivering, while the corresponding figure in the control group was 20.59%.

An important aspect to consider in the interpretation of evidence is the high heterogeneity in the meta-analysis of intra-operative core temperatures. However, there was no heterogeneity noted in the meta-analysis of shivering and intra-operative hypothermia. One reason for the high heterogeneity could be the variable characteristics of study participants, operation theatre protocols, baseline temperature of participants, temperature of the operation theatre, type of blankets used, and surgery type. Most of these variables could not be subjected to a subgroup or meta-regression analysis due to lack of data and the small number of studies available for the analysis. However, we conducted a subgroup analysis based on type of surgery only to note similar outcomes for both thoracic and abdominal surgeries. Due to the high inter-study heterogeneity the results of intra-operative core temperatures should be interpreted with caution.

The better control of intraoperative temperature in the prewarming group can be attributed to the prevention of redistribution of hypothermia. The heat inside the body is usually concentrated in the core regions of the head and trunk, and the peripheral parts remain cooler. However, due to the vasodilatory property of general anesthetics, there is a loss of sympathetic tone, and core heat is transferred to the periphery. A rapid shift of heat occurs in the initial hour of surgery with a reduction in temperature by 0.5–1.5°C. Thereupon, the warmer periphery also causes greater heat loss in the cooler environment of the operating room [26]. Prewarming can increase the temperature of the patient’s peripheral regions before the critical first hour of surgery. Additionally, peripheral vasodilation by prewarming can also prevent the redistribution of hypothermia [12]. These mechanisms may, therefore, aid in better intraoperative control and reduced risk of hypothermia and shivering.

Prewarming as an intervention is safe and can be easily executed. Given the adverse effects of intraoperative hypothermia on patient outcomes [3–6], our study results further strengthen the recommendation to routinely implement prewarming in patients undergoing minimally invasive thoracic and abdominal surgeries. The cost of managing a patient in the post-anesthesia care unit translates to 648 euros per hour [27]. While there are no reports on the exact effects of prewarming on post-anesthesia care costs, reduction in hypothermic events and postoperative complications can indirectly reduce hospital costs. The forced air warming systems used in all included studies consist of a disposable device that can be easily applied at different body sites in the pre-anesthesia room either by the nursing staff or operating room assistants. However, compliance with warming systems remains an issue. A survey conducted in Asia-Pacific hospitals has shown that only 44% of clinicians employ intraoperative warming, while only 24% routinely use prewarming [28]. Busy operating rooms, lack of monitoring, limited space, and inadequate equipment are certainly limitations of the universal application of prewarming. However, given the evidence derived from this study, prewarming must be incorporated into routine operating room practice to improve patient outcomes.

The strength of this review lies in the incorporation of data only from RCTs and focusing on a similar surgical profile. A detailed analysis of intraoperative temperature measurements, hypothermia, and shivering is presented to guide clinical practice.

Nevertheless, our study has some limitations. The number of available studies was limited. The earlier RCTs had a very small sample size which reduced the statistical power of the review. Differences in data reporting led to a further reduction in the number of studies in each meta-analysis. While all studies used the forced air warming system, the differences in prewarming time, type and duration of surgical procedure, and other heat control measures are important confounders that can alter the outcomes. Such differences could have led to high interstudy heterogeneity in the meta-analysis. Lastly, the review was unable to examine the effects of prewarming on postoperative complications due to limited data. Further studies are needed to examine this outcome.

## Conclusions

Moderate to low-quality evidence shows that prewarming combined with intraoperative warming, as compared to intraoperative warming alone, can improve intraoperative temperature control and reduce the risk of hypothermia and shivering in patients undergoing thoracoscopic and laparoscopic procedures.

## Supporting information

S1 Table Search strategy of all databases. (DOCX)

S2 Table PRISMA reporting guidelines. (DOCX)

S3 Table List of excluded studies. (DOCX)

S4 Table GRADE assessment of evidence. (DOCX)
