# Supplementary material for: Comparison of prewarming plus intraoperative warming with intraoperative warming alone in patients undergoing minimally invasive thoracic or abdominal surgery: A systematic review and meta-analysis
Source: PLoS One. 2024 Sep 16;19(9):e0310096. doi: 10.1371/journal.pone.0310096 (PMC11404788; doi:10.1371/journal.pone.0310096)
Supplement: S1 Table — (DOCX) [file pone.0310096.s001.docx]

Supplementary Table 1: Search strategy

For PubMed:

(((thoracoscopic) OR (laparoscopic)) OR (minimally invasive surgery)) AND ((prewarming) OR (hypothermia))

For Embase:

1. 'thoracoscopic surgery'/exp OR 'thoracoscopic surgery'

2. 'laparoscopic surgery'

3. 'minimally invasive surgery'

4. #1 OR #2 OR #3

5. ‘prewarming’

6. 'hypothermia'

7. #5 OR #6

8. #4 AND #7

For CENTRAL

#1 (prewarming) OR (hypothermia)

#2 (thoracoscopic) OR (laparoscopic) OR (minimally invasive surgery)

#3. #1 AND #2

For Web of Science

(((thoracoscopic) OR (laparoscopic)) OR (minimally invasive surgery)) AND ((prewarming) OR (hypothermia))
