# Supplementary material for: Comparison of prewarming plus intraoperative warming with intraoperative warming alone in patients undergoing minimally invasive thoracic or abdominal surgery: A systematic review and meta-analysis
Source: PLoS One. 2024 Sep 16;19(9):e0310096. doi: 10.1371/journal.pone.0310096 (PMC11404788; doi:10.1371/journal.pone.0310096)
Supplement: S3 Table — (DOCX) [file pone.0310096.s003.docx]

Supplementary Table 3: List of excluded studies

| Excluded study | Reason |
| --- | --- |
| Becarra et al[1] | Not randomized controlled trial |
| Munday et al[2] | Not on minimally invasive surgery |
| Thiel et al[3] | Not on minimally invasive surgery |
| Chung et al[4] | Not on minimally invasive surgery |
| Rowley et al[5] | Not on minimally invasive surgery |
| Mohan et al[6] | Not on minimally invasive surgery |
| Fettes et al[7] | Not on minimally invasive surgery |
| Jun et al[8] | Not on minimally invasive surgery |
| De Bernardis et al[9] | Not on minimally invasive surgery |
| Cho et al[10] | Not on minimally invasive surgery |
| Luo et al[11] | Not on prewarming |
| Okgun et al[12] | Not on prewarming |
| Alkan et al[13] | Not on prewarming |
| Su et al[14] | Not on prewarming |
| Nguyen et al[15] | Not on prewarming |

References

1. Becerra Á, Valencia L, Ferrando C, Villar J, Rodríguez-Pérez A. Prospective observational study of the effectiveness of prewarming on perioperative hypothermia in surgical patients submitted to spinal anesthesia. Sci Rep. 2019;9: 16477. doi:10.1038/s41598-019-52960-6

2. Munday J, Osborne S, Yates P, Sturgess D, Jones L, Gosden E. Preoperative Warming Versus no Preoperative Warming for Maintenance of Normothermia in Women Receiving Intrathecal Morphine for Cesarean Delivery: A Single-Blinded, Randomized Controlled Trial. Anesth Analg. 2018;126: 183–189. doi:10.1213/ANE.0000000000002026

3. Thiel B, Mooijer BC, Kolff-Gart AS, Kerklaan BM, Poolman RW, de Haan P, et al. Is preoperative forced-air warming effective in the prevention of hypothermia in orthopedic surgical patients? A randomized controlled trial. J Clin Anesth. 2020;61: 109633. doi:10.1016/j.jclinane.2019.109633

4. Chung SH, Lee B-S, Yang HJ, Kweon KS, Kim H-H, Song J, et al. Effect of preoperative warming during cesarean section under spinal anesthesia. Korean J Anesthesiol. 2012;62: 454–60. doi:10.4097/kjae.2012.62.5.454

5. Rowley B, Kerr M, Van Poperin J, Everett C, Stommel M, Lehto RH. Perioperative Warming in Surgical Patients: A Comparison of Interventions. Clin Nurs Res. 2015;24: 432–41. doi:10.1177/1054773814535428

6. Mohan C, Madhusudhana R. Effects of Pre-warming and Co-warming in Preventing Intraoperative Hypothermia. Cureus. 2023;15: e35132. doi:10.7759/cureus.35132

7. Fettes S, Mulvaine M, Van Doren E. Effect of preoperative forced-air warming on postoperative temperature and postanesthesia care unit length of stay. AORN J. 2013;97: 323–8. doi:10.1016/j.aorn.2012.12.011

8. Jun J-H, Chung MH, Kim EM, Jun I-J, Kim JH, Hyeon J-S, et al. Effect of pre-warming on perioperative hypothermia during holmium laser enucleation of the prostate under spinal anesthesia: a prospective randomized controlled trial. BMC Anesthesiol. 2018;18: 201. doi:10.1186/s12871-018-0668-4

9. de Bernardis RCG, Siaulys MM, Vieira JE, Mathias LAST. Perioperative warming with a thermal gown prevents maternal temperature loss during elective cesarean section. A randomized clinical trial. Brazilian J Anesthesiol. 2016;66: 451–5. doi:10.1016/j.bjane.2014.12.007

10. Cho J, Lee J-M, Kim K-M, Yon JH, Lee HS, Jun I-J. Effect of 10 Minutes of Prewarming and Prewarmed Intravenous Fluid Administration on the Core Temperature of Patients Undergoing Transurethral Surgery under General Anesthesia. Int J Med Sci. 2024;21: 1–7. doi:10.7150/ijms.88943

11. Luo J, Zhou L, Lin S, Yan W, Huang L, Liang S. Beneficial effect of fluid warming in elderly patients with bladder cancer undergoing Da Vinci robotic-assisted laparoscopic radical cystectomy. Clinics (Sao Paulo). 2020;75: e1639. doi:10.6061/clinics/2020/e1639

12. Okgün Alcan A, Aygün H, Kurt C. Resistive Warming Mattress, Forced-Air Warming System, or a Combination of the Two in the Prevention of Intraoperative Inadvertent Hypothermia: A Randomized Trial. J perianesthesia Nurs Off J Am Soc PeriAnesthesia Nurses. 2023;38: 611–615. doi:10.1016/j.jopan.2022.11.007

13. Alkan Bayburt F, Meyanci Koksal G, Bulut A, Sengul I. Intraoperative Patient Warming Instead of Gas on the Management of Postoperative Pain in Laparoscopic Colectomy and Cholecystectomy: A Randomized Controlled Trial. Cureus. 2024;16: e57989. doi:10.7759/cureus.57989

14. Su S-F, Nieh H-C. Efficacy of forced-air warming for preventing perioperative hypothermia and related complications in patients undergoing laparoscopic surgery: A randomized controlled trial. Int J Nurs Pract. 2018;24: e12660. doi:10.1111/ijn.12660

15. Nguyen NT, Fleming NW, Singh A, Lee SJ, Goldman CD, Wolfe BM. Evaluation of core temperature during laparoscopic and open gastric bypass. Obes Surg. 2001;11: 570–5. doi:10.1381/09608920160557039
