# Supplementary material for: Comparison of prewarming plus intraoperative warming with intraoperative warming alone in patients undergoing minimally invasive thoracic or abdominal surgery: A systematic review and meta-analysis
Source: PLoS One. 2024 Sep 16;19(9):e0310096. doi: 10.1371/journal.pone.0310096 (PMC11404788; doi:10.1371/journal.pone.0310096)
Supplement: S4 Table — (DOCX) [file pone.0310096.s004.docx]

**Supplementary Table 4: GRADE assessment of evidence**

| **Certainty assessment** | | | | | | | **№ of patients** | | **Effect** | | **Certainty** | **Importance** |
| --- | --- | --- | --- | --- | --- | --- | --- | --- | --- | --- | --- | --- |
| **№ of studies** | **Study design** | **Risk of bias** | **Inconsistency** | **Indirectness** | **Imprecision** | **Other considerations** | **Prewarming + intraoperative warming** | **Intraoperative warming** | **Relative (95% CI)** | **Absolute (95% CI)** |  |  |
| **intraoperative core temperature - T1** | | | | | | | | | | | | |
| 4 | randomised trials | serious^a^ | not serious | not serious | not serious | none | 121 | 121 | - | MD **0.08 higher** (0.09 lower to 0.24 higher) | ⨁⨁⨁◯ Moderate |  |
| **intraoperative core temperature - T2** | | | | | | | | | | | | |
| 5 | randomised trials | serious^a^ | not serious | not serious | not serious | none | 169 | 170 | - | MD **0.32 higher** (0.15 higher to 0.5 higher) | ⨁⨁⨁◯ Moderate |  |
| **intraoperative core temperature - T3** | | | | | | | | | | | | |
| 6 | randomised trials | serious^a^ | not serious | not serious | not serious | none | 178 | 178 | - | MD **0.37 higher** (0.24 higher to 0.5 higher) | ⨁⨁⨁◯ Moderate |  |
| **intraoperative core temperature - T4** | | | | | | | | | | | | |
| 3 | randomised trials | serious^a^ | not serious | not serious | not serious | none | 96 | 95 | - | MD **0.34 higher** (0.12 higher to 0.56 higher) | ⨁⨁⨁◯ Moderate |  |
| **intraoperative core temperature- T5** | | | | | | | | | | | | |
| 5 | randomised trials | serious^a^ | not serious | not serious | not serious | none | 169 | 170 | - | MD **0.35 higher** (0.25 higher to 0.45 higher) | ⨁⨁⨁◯ Moderate |  |
| **Minimum intra-operative temperature** | | | | | | | | | | | | |
| 2 | randomised trials | serious^a^ | not serious | not serious | serious^b^ | none | 119 | 156 | - | MD **0.62 higher** (0.07 higher to 1.17 higher) | ⨁⨁◯◯ Low |  |
| **Shivering** | | | | | | | | | | | | |
| 5 | randomised trials | serious^a^ | not serious | not serious | serious^b^ | none | 8/170 (4.7%) | 35/170 (20.6%) | **OR 0.18** (0.08 to 0.43) | **161 fewer per 1,000** (from 186 fewer to 106 fewer) | ⨁⨁◯◯ Low |  |
| **Hypothermia** | | | | | | | | | | | | |
| 3 | randomised trials | serious^a^ | not serious | not serious | serious^b^ | none | 14/109 (12.8%) | 43/110 (39.1%) | **OR 0.20** (0.10 to 0.41) | **277 fewer per 1,000** (from 331 fewer to 183 fewer) | ⨁⨁◯◯ Low |  |

**CI:** confidence interval; **MD:** mean difference; **OR:** odds ratio

#### Explanations

a. High risk of bias in several studies

b. wide 95% confidence intervals
